# How do cardiologists select patients for dual antiplatelet therapy continuation beyond 1 year after a myocardial infarction? Insights from the EYESHOT Post‐MI Study

**DOI:** 10.1002/clc.23262

**Published:** 2019-08-31

**Authors:** Leonardo De Luca, Furio Colivicchi, Jennifer Meessen, Massimo Uguccioni, Federico Piscione, Paola Bernabò, Gerardina Lardieri, Antonino Granatelli, Domenico Gabrielli, Michele M. Gulizia, on behalf of the EYESHOT Post‐MI Investigators, F. Piscione, A. Silverio, R.M. Benvenga, F. Mascia, A. Fusco, S. Cicala, L. Oltrona Visconti, B. Marinoni, U. Canosi, P. Cirillo, B. Trimarco, F. Ziviello, D. Grosseto, M. Menozzi, D. Mezzena, C. Mauro, A. Sasso, A. Bellis, P. Calabrò, F. Gragnano, A. Cesaro, V. Venturelli, V. Porretta, N. Borrelli, C. Indolfi, S. De Rosa, D. Torella, N. Morici, P. Bernabò, M. Molfese, F. Della Rovere, G. Lardieri, T. Caiffa, G. Moretto, G Grippo, E. Di Vincenzo, A. Granatelli, L. Lucisano, M. Pennacchi, G. Lardieri, T. Caiffa, G. Geraci, N. Sanfilippo, A. Ledda, A. Di Lenarda, A. Cherubini, G. Russo, F. Piemonte, A. Di Donato, A. Carraturo, B. Villari, Q. Ciampi, C. Contaldi, V. Pacher, E. Corrada, D. Cattani, D. Nassiacos, S. Meloni, B. Barco, R. Bonmassari, A. Bertoldi, F. Tedoldi, M. Cannone, G. Valenti, R.L. Musci, P. Caldarola, N. Locuratolo, L. Sublimi Saponetti, L. Gentili, C. Maiandi, M. Caputo, C.A. Capparuccia, T. Tonella, F.M. Massari, A. Lupi, M. Tessitori, M. Montano, A. Scaglione, A. Torri, G. Tortorella, A. Navazio, R. Cemin, L. Latina, D. Briguglia, R. Marino, S. Scalvini, E. Zanelli, V. Paganini, G. Riboni, E. Leiballi, A. Della Mattia, F. Imperadore, M. Tespili, G. Santangelo, U. Parravicini, P. Dellavesa, R. Testa, E. Venturini, M. Feola, M. Testa, V. Crisci, M. Tramontana, L. Robiglio, F. Varbella, I. Meynet, A. Galati, A. Maddaluna, C. Bilato, I. Loddo, G. Licciardello, L. Cassaniti, M. Scherillo, D. Formigli, L. Marullo, L. Chianese, C. Paolillo, A.P.A. De Santis, N.D. Brunetti, D. Bottigliero, R. Della Bona, M.B. Giannico, R. Tramarin, S. Lucibello, G.P. Perna, M. Marini, A.R. Colavita, M.M. Gulizia, G.M. Francese, M. Mariani, F. Collauto, M. D'Urbano, R. Naio, G. Andò, F. Saporito, E.M. Assanelli, A. Cabiati, A. Crivaro, S. Alberti, I. Marchese, T. Nejat, S. Refice, F. Colivicchi, A. Aiello, A. Galati, G.R. Cristinziani, F. Barillà, R Iorio, G. Mascelli, S.N. Tartaglione, G. Di Chiara, D. D'Andrea, R. Antonicelli, G. Malatesta, C. Di Mario, A. Mattesini, L. Tramontana, S. Conti, L. Sommariva, A. Celestini, F. Amico, S. Giubilato, A.F. Amico, M. De Filippis, G.F. Pasini, M Triggiani, V. Ferrara, S. Cappetti, S. Carugo, S. Lucreziotti, M. Persico, G. Gizzi, T. Cipolla, A. Caronia, E. Buia, P. Pastori, M. Scarpignato, E. Biscottini, F. Poletti, C. Vimercati, R. Pirola, E. Barbieri, C Dugo, N. De Cesare, M.L. De Benedictis, A. Ruggeri, C. Campana, S. Bonura, C. Vigna, N. Marchese, N.G. Partesana, P. Bandini, G. Farinola, D. Santoro, F. Cassadonte, F. Calabrò, M. Sansoni, M.G. Abrignani, F. Bonura, D. Gabrielli, M. Benvenuto, A. Liso, T. Passero, I. Mori, B. Pozzoni, F. Prati, M.L. Finocchiaro, N. Tufano, B. Miserrafiti, V. Lacquaniti, F. Del Piccolo, B. Mohamad, M.T. Spinnler, V. Bovolo, E. Rebulla, M. Pieri, L. Paloscia, D. Di Clemente, G. Mazzucco, A. Micanti, P. Peci, O. Ornago, F. Proietti, M. Michisanti, A. Reverzani, A. Donatini, P. Costa, S. Russo, E. Franceschini Grisolia, L. Mario, F. Di Palma, F. Dell'Aquila, A. Maestroni, SI Caico, G. De Caro, L. Attianese, S. Perotti, V. Cotti Cometti, D. Astengo, A. Navazio, E. Guerri, D. Cianflone, F. Maranta, N. Esposito, M. Malvezzi Caracciolo D'Aquino, L. Caliendo, C. Ricci, C.P. Ceruso, S. Lanteri, R. Serdoz, E. Bruno, C. De Matteis, C. Campagnuolo, M.A. Ammirati, V.M. Corrado, M.A. Amado Eleas, L. Fattore, C. Ippoliti, G. Turiano, C. Piergentili, S.I. Caico, F. Chiarella, P. Capogrosso, M. Perotti, S. Di Marco, G. Sibilio, L. Di Lorenzo, A. Aurelio, A.B. Ramondo, D. Zanna, C. Cernetti, G. Napolitano, S. Negroni, N. Alessandri, F. Rigo, F. Giusti, G. Casu, A. Vicentini, G. Calculli, M.S. Fera, G.V. Lettica, G. Vagheggini, A. Pitì, A. Porfidia, A. Di Leo, A. Ravera, E. Ciotta, S. Saccà, O. Silvestri, S. Isidori, P. Natali, M. Anselmi, L. Testa, A. Antonelli, E. Tavasci, G. Furgi, A. Lavorgna, N. Gasparetto, T. Bisceglia

**Affiliations:** ^1^ Division of Cardiology S. Giovanni Evangelista Hospital Tivoli Italy; ^2^ Division of Cardiology S. Filippo Neri Hospital Rome Italy; ^3^ Department of Cardiovascular Research Istituto di Ricerche Farmacologiche Mario Negri Milan Italy; ^4^ Division of Cardiology Azienda Ospedaliera S. Camillo‐Forlanini Rome Italy; ^5^ Division of Preventive Cardiology SS Giovanni di Dio e Ruggi d'Aragona Hospital, University of Salerno Fisciano Italy; ^6^ Division of Cardiology Ospedali Galliera Genoa Italy; ^7^ Division of Cardiology Ospedale di Gorizia e Monfalcone Monfalcone Salerno Italy; ^8^ Division of Cardiology Augusto Murri Hospital Fermo Italy; ^9^ Division of Cardiology Garibaldi‐Nesima Hospital Catania Italy; ^10^ Heart Care Foundation Florence Italy; ^11^ Salerno; ^12^ Caserta, A.O.S. Anna e S. Sebastiano; ^13^ Pavia, Fondazione IRCCS Pol. S. Matteo; ^14^ Napoli, AOU Federico II; ^15^ Rimini; ^16^ Napoli, AORN Cardarelli, Cardiologia c/UTIC; ^17^ Napoli, AORN Osp. dei Colli‐PO V. Monaldi; ^18^ Roma, S. Pertini; ^19^ Catanzaro, Materdomini; ^20^ Milano, Niguarda, Cardiologia; ^21^ Genova, Galliera; ^22^ Monfalcone; ^23^ Prato; ^24^ Tivoli; ^25^ Gorizia; ^26^ Palermo, Villa Sofia‐Cervello; ^27^ Trieste; ^28^ Frattamaggiore; ^29^ Benevento, Sacro Cuore di Gesù FBF; ^30^ Rozzano; ^31^ Saronno; ^32^ Trento; ^33^ Andria; ^34^ Bari, San Paolo; ^35^ Brescia; ^36^ Chieti; ^37^ Milano, Maggiore Policlinico; ^38^ Verbania; ^39^ Milano, S.M. Nascente; ^40^ Reggio Emilia; ^41^ Bolzano; ^42^ Castellanza; ^43^ Lumezzane; ^44^ Montescano; ^45^ Pordenone; ^46^ Rovereto; ^47^ Seriate; ^48^ Borgomanero; ^49^ Cecina; ^50^ Fossano; ^51^ Lentini; ^52^ Lido di Camaiore; ^53^ Rivoli; ^54^ Roma, Villa Betania; ^55^ Arzignano; ^56^ Augusta; ^57^ Benevento, G. Rummo; ^58^ Castel Volturno; ^59^ Corato; ^60^ Foggia; ^61^ Roma, Casilino; ^62^ San Donato Milanese, IRCCS, Cardiologia Riabilitativa; ^63^ Ancona, Riuniti; ^64^ Campobasso; ^65^ Catania, Garibaldi‐Nesima; ^66^ Cuggiono; ^67^ Magenta; ^68^ Messina; ^69^ Milano, Monzino; ^70^ Paola; ^71^ Rieti; ^72^ Roma, Clinica Città di Roma; ^73^ Roma, P.O.S. Filippo Neri, Cardiologia e UTIC; ^74^ Roma, P.O.S. Filippo Neri, Card. Riab.‐P.O. Salus; ^75^ Roma, Umberto Primo; ^76^ Sanremo; ^77^ Santa Maria Capua Vetere; ^78^ Ancona, INRCA; ^79^ Firenze; ^80^ Spoleto; ^81^ Viterbo; ^82^ Catania, Cannizzaro; ^83^ Galatina; ^84^ Gavardo; ^85^ Manfredonia; ^86^ Milano, San Paolo; ^87^ San Benedetto Del Tronto; ^88^ Cefalù; ^89^ Fidenza; ^90^ Foligno; ^91^ Legnano; ^92^ Milano, Niguarda, Cardiologia; ^93^ Negrar; ^94^ Osio Sotto; ^95^ Reggio Calabria, Madonna della Consolazione; ^96^ San Fermo della Battaglia; ^97^ San Giovanni Rotondo; ^98^ Sondalo; ^99^ Cassano delle Murge; ^100^ Catanzaro, Pugliese; ^101^ Empoli; ^102^ Erice; ^103^ Fermo; ^104^ Lecce; ^105^ Milano, CTO; ^106^ Roma, S.G. Addolorata; ^107^ Sorrento; ^108^ Melito di Porto Salvo; ^109^ Mestre, San Marco; ^110^ Moncalieri; ^111^ Palermo, Casa di Cura Candela; ^112^ Pescara; ^113^ Piossasco; ^114^ Ponte San Pietro; ^115^ Roma, Aurelia Hospital; ^116^ Scandiano; ^117^ Avola; ^118^ Belluno; ^119^ Boscotrecase; ^120^ Busto Arsizio; ^121^ Castellammare di Stabia; ^122^ Esine; ^123^ Genova, Padre Antero Micone; ^124^ Guastalla; ^125^ Milano, S. Raffaele; ^126^ Napoli, Fond. Evangelica Betania; ^127^ Nola; ^128^ Reggio Calabria, Bianchi Melacrino Morelli; ^129^ Roma, S. Pietro FBF; ^130^ San Felice a Cancello; ^131^ San Giuseppe Vesuviano; ^132^ Arco; ^133^ Aversa; ^134^ Avezzano; ^135^ Conegliano; ^136^ Feltre; ^137^ Gallarate; ^138^ Genova, S. Martino; ^139^ Napoli, S.G. Bosco; ^140^ Pavia, ICS Maugeri SPA Società Benefit; ^141^ Pescia; ^142^ Pozzuoli; ^143^ Sessa Aurunca; ^144^ Taranto; ^145^ Vicenza; ^146^ Bari, Policlinico; ^147^ Castelfranco Veneto; ^148^ Giugliano in Campania; ^149^ Imola; ^150^ Latina; ^151^ Mestre, Dell'Angelo; ^152^ Molfetta; ^153^ Nuoro; ^154^ Peschiera Del Garda; ^155^ Policoro; ^156^ Pomezia; ^157^ Vittoria; ^158^ Volterra; ^159^ Bergamo; ^160^ Caserta, Villa del Sole; ^161^ Ciriè; ^162^ Ivrea; ^163^ Licata; ^164^ Mirano; ^165^ Napoli, AORN Cardarelli, Cardiologia Generale c/Riabilitazione; ^166^ Piombino; ^167^ S. Omero; ^168^ San Bonifacio; ^169^ San Donato Milanese, IRCCS, Cardiologia c/UTIC; ^170^ Sesto San Giovanni; ^171^ Sondrio; ^172^ Telese; ^173^ Teramo; ^174^ Treviso; ^175^ Udine

**Keywords:** clopidogrel, dual antiplatelet therapy, percutaneous coronary intervention, post‐MI, secondary prevention, ticagrelor

## Abstract

**Background:**

Current guidelines suggest to consider dual antiplatelet therapy (DAPT) continuation for longer than 12 months in selected patients with myocardial infarction (MI).

**Hypothesis:**

We sought to assess the criteria used by cardiologists in daily practice to select patients with a history of MI eligible for DAPT continuation beyond 1 year.

**Methods:**

We analyzed data from the EYESHOT Post‐MI, a prospective, observational, nationwide study aimed to evaluate the management of patients presenting to cardiologists 1 to 3 years from the last MI event.

**Results:**

Out of the 1633 post‐MI patients enrolled in the study between March and December 2017, 557 (34.1%) were on DAPT at the time of enrolment, and 450 (27.6%) were prescribed DAPT after cardiologist assessment. At multivariate analyses, a percutaneous coronary intervention (PCI) with multiple stents and the presence of peripheral artery disease (PAD) resulted as independent predictors of DAPT continuation, while atrial fibrillation was the only independent predictor of DAPT interruption for patients both at the second and the third year from MI at enrolment and the time of discharge/end of the visit.

**Conclusions:**

Risk scores recommended by current guidelines for guiding decisions on DAPT duration are underused and misused in clinical practice. A PCI with multiple stents and a history of PAD resulted as the clinical variables more frequently associated with DAPT continuation beyond 1 year from the index MI.

## 1 INTRODUCTION

Current guidelines suggest that continuation of dual antiplatelet therapy (DAPT) for longer than 12 months should be considered in patients with myocardial infarction (MI) who have tolerated DAPT without bleeding complications.^1^, ^2^ This recommendation is based on recent randomized clinical trials suggesting that DAPT prolongation reduces the rate of recurrent ischemic events in post‐MI patients, compared with aspirin alone.^3^, ^4^ However, these benefits come at the cost of increased risk of bleeding, raising the question about how to identify the ideal patient profile who could safely prolong DAPT.^1^, ^2^, ^5^, ^6^ Although predictors of DAPT interruption have been already identified in large international registries,^7^, ^8^, ^9^ specific appraisals underlying the decision on the extension of DAPT have been poorly investigated.^10^, ^11^

Using data from the EYESHOT (EmploYEd antithrombotic therapies in patients with acute coronary Syndromes HOspitalized in iTaly) Post‐MI study^12^ we sought to evaluate the criteria used by cardiologists in daily clinical practice for selecting post‐MI patients eligible for DAPT continuation beyond 1 year.

## 2 METHODS

The methods used for the EYESHOT Post‐MI study have been described previously.^12^ Briefly, it was a prospective, observational, nationwide registry of consecutive patients with a prior MI managed by cardiologists. All patients admitted in cardiology units and/or ambulatory clinics during a period of 3 months with a documented history of presumed spontaneous MI event (non‐ST‐elevation, NSTEMI or ST‐elevation‐MI, STEMI) occurred between 1 and 3 years before enrolment have been included.^12^ Patients were enrolled at the beginning of outpatient or day‐hospital visit or at hospital admission. Physicians were asked to report medications at enrolment and at the end of the visit or hospital discharge.

The Italian Association of Hospital Cardiologists (ANMCO) designed, endorsed, and conducted the study. All patients were informed of the nature and aims of the study and asked to sign an informed consent for the anonymous management of their individual data. Local Institutional Review Boards (IRB) approved the study protocol according to the current Italian rules. Data were collected in different periods of consecutive 3 months in each site between 1 March 2017 and 16 December 2017. Over these periods, 1633 consecutive patients [median 22 (IQR 15‐28) months from MI] were enrolled: 1028 (63.0%) presenting to a cardiologist during the second [median 17 (IQR 14‐21) months] and 605 (37.0%) during the third year from MI [median 30 (IQR 27‐33) months].^12^ The study has been carried out in 165 cardiology centers. Most of the patients were enrolled during outpatient or day‐hospital visits (84%) and the remaining during hospital admissions (16%).^12^

Data were collected using a web‐based, electronic case report form with the central database located at the ANMCO Research Center. By using a validation plan, integrated in the data entry software, data were checked for missing or contradictory entries and values out of the normal range.

### 2.1 Statistical analysis

Categorical variables are presented as number and percentages and compared by the *χ* ^2^ test. Normally distributed, continuous variables are presented as mean and SD (SD) and compared by means of *t* test, while not normally distributed variables are reported as median and interquartile range (IQR)and assessed by the Mann‐Whitney *U* test.

The study cohort was stratified according to the treatment with DAPT at the time of enrolment.

Clinically relevant variables were included in a multivariable model (logistic regression), to identify the independent predictors of DAPT assumption at the time of enrolment and after hospital discharge from a cardiology ward or at the end of the visit by cardiologist among patients in the second and the third year from the index MI. The variables included in the logistic model were: age, gender, diabetes mellitus, renal insufficiency, peripheral artery disease (PAD), history of major bleeding events or surgery, heart failure, atrial fibrillation, presence of symptoms, prior revascularization, number of stent implanted (≤2 vs >2), type of last myocardial infarction (STEMI vs NSTEMI), type of enrolment (hospital admission vs outpatient visit). A *P* value < .05 was considered statistically significant. All tests were two‐sided. Analyses were performed with SPSS system software, version 24.

## 3 RESULTS

**Figure 1:**
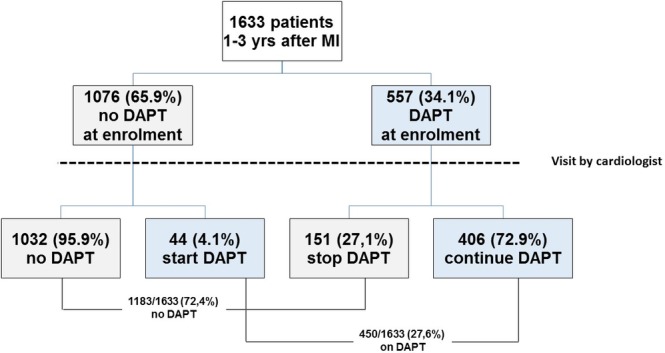
Patients flow‐chart

Out of the 1633 post‐MI patients enrolled, 557 (34.1%) were on DAPT at the time of enrolment (413 in the second and 144 in the third year from the index MI). At the time of discharge/end of the visit, 450 (27.6%) were prescribed DAPT (317 in the second and 133 in the third year from the index MI) (Figure 1). At the end of the visit/hospital discharge a DAPT was initiated by cardiologists in 4% (44/1076) of patients, while in 27% of cases (151/557) DAPT was withdrawn (Figure 1). Out of the 151 patients who interrupted DAPT after the visit by a cardiologist, only five (3.3%) withdrew at least one antiplatelet agent for a planned major surgery; in the remaining 146 patients DAPT was interrupted for clinical reasons.

Baseline characteristics of patients on DAPT vs those not receiving DAPT at enrolment are shown in Table 1. Patients on DAPT were on average younger (64 vs 66 years, *P* = .002) and had a shorter time since index MI (20 vs 23 months, *P* < .001). In addition, patients on DAPT had less frequently atrial fibrillation (6.8% vs 15.9%, *P* < .0001) but did have a higher incidence of PAD compared to patients without DAPT at enrolment (9.3% vs 5.6%, *P* = .005).

**Table 1:** Clinical characteristics and laboratory variables of patients on DAPT and those not receiving DAPT at the time of enrolment

	no DAPT N = 1076	DAPT N = 557	*P* value
Age, mean ± SD	66 ± 11	64 ± 12	.002
Age ≥ 75 years, n (%)	258 (24.0)	100 (18.0)	.005
Males, n (%)	857 (79.6)	457 (82.0)	.263
Type MI, n (%)			.076
STEMI	561 (52.1)	264 (47.4)	
NSTEMI	515 (47.9)	293 (52.6)	
Months since MI, mean ± SD	23 ± 7	20 ± 7	<.0001
BMI, mean ± SD	27.2 ± 4	27.2 ± 4	.969
Active smokers, n (%)	199 (18.5)	109 (19.6)	.829
Diabetes mellitus, n (%)	293 (27.2)	168 (30.2)	.223
Hypertension,^a^ n (%)	832 (77.3)	451 (81.0)	.098
Hypercholesterolemia, n (%)	790 (73.4)	422 (75.8)	.311
Atrial fibrillation, n (%)	171 (15.9)	38 (6.8)	<.0001
Renal dysfunction,^b^ n (%)	124 (11.5)	79 (14.2)	.133
Peripheral artery disease,^c^ n (%)	60 (5.6)	52 (9.3)	.005
COPD, n (%)	120 (11.2)	67 (12.0)	.623
Stroke/TIA, n (%)	48 (4.5)	20 (3.6)	.436
Major bleeding,^d^ n (%)	39 (3.6)	11 (2.0)	.070
Heart failure, n (%)	159 (14.8)	94 (16.9)	.279
PCI with >2 stent, n (%)	184 (17.1)	171 (30.7)	<.0001
Prior CABG, n (%)	111 (10.3)	60 (10.8)	.798
Ejection fraction, %, mean ± SD	48 ± 18	47 ± 18	.066
Systolic blood pressure, mmHg, mean ± SD	131 ± 18	129 ± 17	.015
Heart rate, bpm, mean ± SD	67 ± 12	67 ± 11	.114
Hemoglobin, g/dL, median [IQR]	14.0 [12.6‐15.0]	13,8 [12.3‐14.9]	.303
Creatinine, mg/dL, median [IQR]	1.0 [0.8‐1.2]	1,0 [0.8‐1.2]	.503
Total cholesterol, mg/dL, median [IQR]	140 [122‐162]	139 [122‐165]	.876
LDL cholesterol, mg/dL, median [IQR]	72 [56‐91]	70 [58‐90]	.963
Triglycerides, mg/dL, median [IQR]	103 [79‐141]	110 [83‐155]	.0049
Glycemia, mg/dL, median [IQR]	103 [92‐119]	103 [93‐122]	.573

a SBP≥140 mmHg or diastolic blood pressure ≥ 90 mmHg or use of blood pressure lowering drugs.

b Dialysis, history of renal transplant or creatinine levels >1.5 mg/dL.

c History of claudication; amputation for arterial insufficiency; aorta‐iliac occlusive disease reconstruction surgery; peripheral vascular bypass surgery, angioplasty, or stent; documented abdominal aortic aneurysm, aneurysm repair or stent; and documented positive non‐invasive testing such as abnormal ankle‐brachial index or pulse volume recording.

d Fatal bleeding^,^ or clinically evident bleeding with hemoglobin reduction ≥2 g/dL or requiring transfusion or hospitalization.

Abbreviations: BMI, body mass index; COPD, chronic obstructive pulmonary disease; HR, heart rate; LDL, low density lipoprotein; SBP, systolic blood pressure; STEMI, ST‐elevation myocardial infarction; TIA, transient ischemic attack.

PCI with multiple stents implantation was more frequent in patients on DAPT (30.7% vs 17.1%, *P* < .0001). Systolic blood pressure (129 vs 131 mmHg, *P* = .015) was lower in patients on DAPT whereas levels of triglycerides were higher (110 vs 103 mg/dL, *P* = .049) as compared to patients without DAPT (Table 1).

Among patients receiving DAPT, the most frequently used combination was aspirin and clopidogrel for both patients in the second and third year from MI (Table 2).

**Table 2:** Type of P2Y12 inhibitor associated with aspirin at enrolment and after cardiologist' assessment according to the time from last MI (12‐24 months vs 24‐36 months)

		Time from MI	
		12‐24 months	25‐36 months
DAPT at enrolment	Clopidogrel	188 (45.5%)	114 (79.2%)
N = 557			
	Prasugrel	60 (14.5%)	5 (3.5%)
	Ticagrelor 90 mg bid	143 (34.6%)	21 (14.6%)
	Ticagrelor 60 mg bid	20 (4.8%)	3 (0.2%)
	Ticlopidine	2 (0.5%)	1 (0.1%)
	**Total**	**413**	**144**
DAPT after cardiologist assessment	Clopidogrel	177 (55.8%)	112 (84.2%)
N = 450			
	Prasugrel	27 (8.5%)	2 (1.5%)
	Ticagrelor 90 mg bid	64 (20.2%)	15 (11.3%)
	Ticagrelor 60 mg bid	47 (14.8%)	3 (2.3%)
	Ticlopidine	2 (0.6%)	1 (0.8%)
	**Total**	**317**	**133**

**Figure 2:**
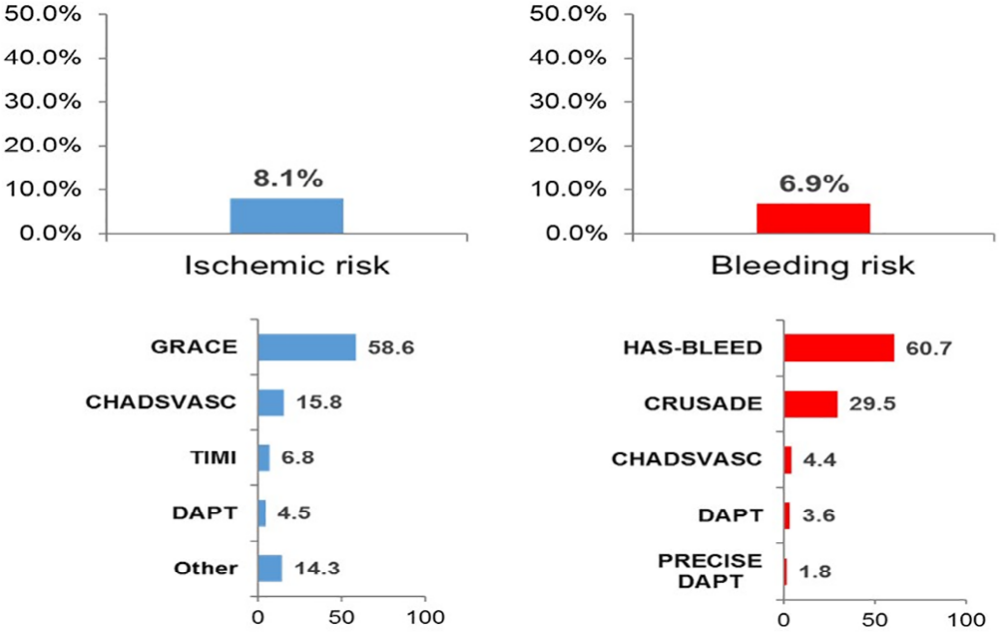
Use of scores for the assessment of ischemic or bleeding risk

Scores for the assessment of ischemic or bleeding risk were used in 8.1% and 6.9% of patients, respectively (Figure 2). The GRACE and the HAS‐BLEED resulted as the risk scores mostly used for the evaluation of ischemic or bleeding risk, respectively (Figure 2). Ischemic risk scores were used in 9.5% of the patients on DAPT and in 7.4% of patients without DAPT (*P* = .153); whereas bleeding risk scores were used in 8.1% and 6.2% of patients on DAPT and without DAPT, respectively (*P* = .179).

**Figure 3:**
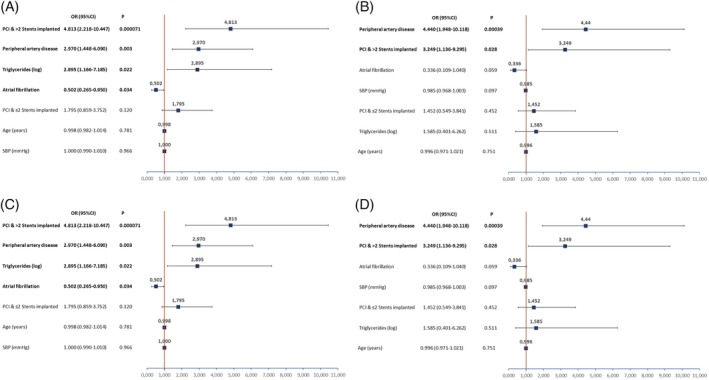
Multivariable analysis on DAPT assumption at the time of enrolment for patients in the second (Panel A) and third (Panel B) year from the last MI and after cardiologist assessment for patients in the second (Panel C) and third (Panel D) year from the last MI

At multivariate analyses, PCI with multiple stents and PAD resulted as independent predictors of DAPT continuation, while atrial fibrillation was the sole independent predictor of DAPT interruption for both patients at the second and the third year from MI at enrolment and the time of discharge/end of the visit (Figure 3).

## 4 DISCUSSION

The present analysis of a nationwide study on consecutive patients managed by cardiologists 1‐3 years after a MI demonstrates that: (a) DAPT was withdrawn in approximately one of three patients enrolled and in less than 5% of cases DAPT was initiated after cardiologist' assessment; (b) Risk scores for the identification of patients who can benefit from DAPT prolongation are mis‐ and underused in clinical practice; (c) Patients with a complex PCI and a history of PAD are those who more frequently continue DAPT beyond 1 year from the index MI.

In post‐MI patients, the cardiovascular risk remains substantially elevated beyond the first year.^13^, ^14^, ^15^ The REACH (REduction of Atherothrombosis for Continued Health) registry showed an incidence of recurrent cardiovascular events of 18% at 4 years in patients with history of MI or stroke.^13^ Accordingly, the APOLLO dataset, which links registries and administrative data, demonstrated that the risk of cardiovascular events in MI survivors is approximately 20% across the first 3 years from MI.^14^, ^15^ In this setting, prolonged DAPT has been shown to be an effective therapeutic strategy to prevent recurrent ischemic events.^1^, ^2^, ^3^, ^4^ Nonetheless, because continued antiplatelet therapy is also associated with increased risk of bleeding, it is necessary to weigh this risk against the potential benefit.^1^, ^2^, ^3^, ^4^ Decisions about duration of DAPT are best made on an individual basis and should integrate several clinical variables. In this regard, novel risk scores have been specifically designed to guide and inform decision making for optimal DAPT duration.^16^ However, most of the frequently used risk scores for assessing ischemic events or major bleedings in our cohort were originally developed and validated for the prediction of events occurring mainly during hospital stay or at short term follow‐up after a MI or in the setting of atrial fibrillation.^17^, ^18^ As a result, the application of these risk scores to decide upon DAPT prolongation seems inappropriate, as only limited data exist exploring their value to guide DAPT duration. On the other hand, risk scores specifically validated for assessing DAPT duration, such as the DAPT or the PRECISE‐DAPT (PREdicting bleeding Complications In patients undergoing Stent implantation and subsEquent Dual Anti Platelet Therapy) scores,^19^, ^20^ have been extremely underused in our registry.

Several studies demonstrated major bleeding events and urgent surgery are the most common reasons for early DAPT interruption.^7^, ^8^, ^9^ However, few have investigated the reasons why cardiologists prolong or interrupt DAPT beyond the first year after a MI.^10^, ^11^ The EPICOR study (long‐term follow‐up of antithrombotic management patterns in acute coronary syndrome patients), conducted in 2010‐2011 in 20 countries, showed that more than half of patients with MI remained on DAPT beyond 12 months in Europe and Latin America.^10^ Subsequently, the TIGRIS registry, conducted in 2013‐2014 in 25 countries, documented DAPT continuation beyond 1 year in 12% of post‐MI patients with high‐risk features enrolled in Europe, 25% in North America and 40% in Asia‐Pacific countries.^11^ In both international registries,^10^, ^11^ the presence of frequent PCI with multiple stenting represented the most important determinants of DAPT continuation 1 year after the index MI. The EYESHOT Post‐MI study differs from the above mentioned registries since we enrolled patients exclusively managed by specialists in a nationwide setting and within the first 3 years from the last MI event, allowing assessment of the different selection criteria for DAPT continuation according to the timing from the first recommended year of DAPT and excluding patients who already interrupted DAPT for major bleeding events, need for major surgery, cultural and/or economic reasons. Nevertheless, even in our analysis, a complex PCI with multiple stents implantation, together with the presence of PAD, resulted as independent predictor of DAPT continuation for both patients in the second and in the third year from MI. In this regard, emerging evidences suggest that the magnitude of the benefit with long‐term DAPT is progressively greater per increase in PCI procedural complexity.^21^, ^22^ Similarly, several international registries and analyses from clinical trial databases have demonstrated an increased risk of recurrent ischemic events among patients with MI and PAD, including those receiving an early and effective coronary revascularization, and recent studies also suggested a survival benefit of DAPT continuation in these patients.^23^

On the other hand, the presence of atrial fibrillation needing oral anticoagulation therapy was an independent predictor of DAPT interruption in our analysis. This finding is in accordance with recent guidelines and consensus documents recommending a shorter duration of DAPT in order to reduce the risk of major bleeding events.^1^, ^2^, ^24^

### 4.1 Study limitations

Our study must be evaluated in the light of the known limitations of observational, cross‐sectional studies. In addition, even if the participating centers were asked to include in the registry all consecutive post‐MI patients, we were not able to verify the enrolment process, due to the absence of administrative auditing. We believe that it is unlikely however that selective enrolment in few sites may have substantially changed the study results. Finally, even if we tried to collect in the CRF all the possible variables responsible for DAPT interruption or continuation, we cannot exclude some reasons have not been gathered.

## 5 CONCLUSIONS

In this contemporary, nationwide, real‐world study on consecutive patients managed by cardiologists 1 to 3 years after a MI, risk scores recommended by current guidelines for guiding decisions on DAPT duration have been used in a small number of cases. A PCI with multiple stents and a history of PAD resulted as the clinical variables more frequently associated with DAPT continuation beyond 1 year from the index MI. These findings may have important implications for educational programs to improve adherence to current guidelines.

## CONFLICT OF INTEREST

L.D.L. reports personal fees from Astra Zeneca and Daiichi Sankyo outside the submitted work. All other authors have reported that no potential conflicts of interest exist with any companies/organizations whose products or services may be discussed in this article.

## Appendix 1

**Steering Committee**

L. De Luca (Chairman), M.M. Gulizia (Co‐Chairman), F. Colivicchi, A. Di Lenarda, D. Gabrielli, G. Geraci, F. Nardi, R. Rossini.

**Coordinating Center**

ANMCO Research Center (A.P. Maggioni, D. Lucci, A. Lorimer, G. Orsini, L. Gonzini, P. Priami).

**Participating Centers and Investigators**

Salerno (F. Piscione, A. Silverio, R.M. Benvenga); Caserta, A.O.S. Anna e S. Sebastiano (F. Mascia, A. Fusco, S. Cicala); Pavia, Fondazione IRCCS Pol. S. Matteo, (L. Oltrona Visconti, B. Marinoni, U. Canosi); Napoli, AOU Federico II (P. Cirillo, B. Trimarco, F. Ziviello); Rimini (D. Grosseto, M. Menozzi, D. Mezzena); Napoli, AORN Cardarelli, Cardiologia c/UTIC (C. Mauro, A. Sasso, A. Bellis); Napoli, AORN Osp. dei Colli‐PO V. Monaldi (P. Calabrò, F. Gragnano, A. Cesaro); Roma, S. Pertini (V. Venturelli, V. Porretta, N. Borrelli); Catanzaro, Materdomini (C. Indolfi, S. De Rosa, D. Torella); Milano, Niguarda, Cardiologia (N. Morici); Genova, Galliera (P. Bernabò, M. Molfese, F. Della Rovere); Monfalcone (G. Lardieri, T. Caiffa, G. Moretto); Prato (G Grippo, E. Di Vincenzo); Tivoli (A. Granatelli, L. Lucisano, M. Pennacchi); Gorizia (G. Lardieri, T. Caiffa); Palermo, Villa Sofia‐Cervello (G. Geraci, N. Sanfilippo, A. Ledda); Trieste (A. Di Lenarda, A. Cherubini, G. Russo); Frattamaggiore (F. Piemonte, A. Di Donato, A. Carraturo); Benevento, Sacro Cuore di Gesù FBF (B. Villari, Q. Ciampi, C. Contaldi); Rozzano (V. Pacher, E. Corrada, D. Cattani); Saronno (D. Nassiacos, S. Meloni, B. Barco); Trento (R. Bonmassari, A. Bertoldi, F. Tedoldi); Andria (M. Cannone, G. Valenti, R.L. Musci); Bari, San Paolo (P. Caldarola, N. Locuratolo, L. Sublimi Saponetti); Brescia (L. Gentili, C. Maiandi); Chieti (M. Caputo, C.A. Capparuccia); Milano, Maggiore Policlinico (T. Tonella, F.M. Massari); Verbania (A. Lupi, M. Tessitori, M. Montano); Milano, S.M. Nascente (A. Scaglione, A. Torri); Reggio Emilia (G. Tortorella, A. Navazio); Bolzano (R. Cemin, L. Latina); Castellanza (D. Briguglia, R. Marino); Lumezzane (S. Scalvini, E. Zanelli); Montescano (V. Paganini, G. Riboni); Pordenone (E. Leiballi, A. Della Mattia); Rovereto (F. Imperadore); Seriate (M. Tespili, G. Santangelo); Borgomanero (U. Parravicini, P. Dellavesa); Cecina (R. Testa, E. Venturini); Fossano (M. Feola, M. Testa); Lentini (V. Crisci, M. Tramontana); Lido di Camaiore (L. Robiglio); Rivoli (F. Varbella, I. Meynet); Roma, Villa Betania (A. Galati, A. Maddaluna); Arzignano (C. Bilato, I. Loddo); Augusta (G. Licciardello, L. Cassaniti); Benevento, G. Rummo (M. Scherillo, D. Formigli); Castel Volturno (L. Marullo, L. Chianese); Corato (C. Paolillo, A.P.A. De Santis); Foggia (N.D. Brunetti, D. Bottigliero); Roma, Casilino (R. Della Bona, M.B. Giannico); San Donato Milanese, IRCCS, Cardiologia Riabilitativa (R. Tramarin, S. Lucibello); Ancona, Riuniti (G.P. Perna, M. Marini); Campobasso (A.R. Colavita); Catania, Garibaldi‐Nesima (M.M. Gulizia, G.M. Francese); Cuggiono (M. Mariani, F. Collauto); Magenta (M. D'Urbano, R. Naio); Messina (G. Andò, F. Saporito); Milano, Monzino (E.M. Assanelli, A. Cabiati); Paola (A. Crivaro, S. Alberti); Rieti (I. Marchese); Roma, Clinica Città di Roma (T. Nejat, S. Refice); Roma, P.O.S. Filippo Neri, Cardiologia e UTIC (F. Colivicchi, A. Aiello); Roma, P.O.S. Filippo Neri, Card. Riab.‐P.O. Salus (A. Galati, G.R. Cristinziani); Roma, Umberto Primo (F. Barillà, R Iorio); Sanremo (G. Mascelli, S.N. Tartaglione); Santa Maria Capua Vetere (G. Di Chiara, D. D'Andrea); Ancona, INRCA (R. Antonicelli, G. Malatesta); Firenze (C. Di Mario, A. Mattesini); Spoleto (L. Tramontana, S. Conti); Viterbo (L. Sommariva, A. Celestini); Catania, Cannizzaro (F. Amico, S. Giubilato); Galatina (A.F. Amico, M. De Filippis); Gavardo (G.F. Pasini, M Triggiani); Manfredonia (V. Ferrara, S. Cappetti); Milano, San Paolo (S. Carugo, S. Lucreziotti); San Benedetto Del Tronto (M. Persico, G. Gizzi); Cefalù (T. Cipolla, A. Caronia); Fidenza (E. Buia, P. Pastori); Foligno (M. Scarpignato, E. Biscottini); Legnano (F. Poletti, C. Vimercati); Milano, Niguarda, Cardiologia (R. Pirola); Negrar (E. Barbieri, C Dugo); Osio Sotto (N. De Cesare, M.L. De Benedictis); Reggio Calabria, Madonna della Consolazione (A. Ruggeri); San Fermo della Battaglia (C. Campana, S. Bonura); San Giovanni Rotondo (C. Vigna, N. Marchese); Sondalo (N.G. Partesana, P. Bandini); Cassano delle Murge (G. Farinola, D. Santoro); Catanzaro, Pugliese (F. Cassadonte); Empoli (F. Calabrò, M. Sansoni); Erice (M.G. Abrignani, F. Bonura); Fermo (D. Gabrielli, M. Benvenuto); Lecce (A. Liso, T. Passero); Milano, CTO (I. Mori, B. Pozzoni); Roma, S.G. Addolorata (F. Prati, M.L. Finocchiaro); Sorrento (N. Tufano); Melito di Porto Salvo (B. Miserrafiti, V. Lacquaniti); Mestre, San Marco (F. Del Piccolo, B. Mohamad); Moncalieri (M.T. Spinnler, V. Bovolo); Palermo, Casa di Cura Candela (E. Rebulla, M. Pieri); Pescara (L. Paloscia, D. Di Clemente); Piossasco (G. Mazzucco, A. Micanti); Ponte San Pietro (P. Peci, O. Ornago); Roma, Aurelia Hospital (F. Proietti, M. Michisanti); Scandiano (A. Reverzani, A. Donatini); Avola (P. Costa, S. Russo); Belluno (E. Franceschini Grisolia, L. Mario); Boscotrecase (F. Di Palma, F. Dell'Aquila); Busto Arsizio (A. Maestroni, SI Caico); Castellammare di Stabia (G. De Caro, L. Attianese); Esine (S. Perotti, V. Cotti Cometti); Genova, Padre Antero Micone (D. Astengo); Guastalla (A. Navazio, E. Guerri); Milano, S. Raffaele (D. Cianflone, F. Maranta); Napoli, Fond. Evangelica Betania (N. Esposito, M. Malvezzi Caracciolo D'Aquino); Nola (L. Caliendo, C. Ricci); Reggio Calabria, Bianchi Melacrino Morelli (C.P. Ceruso, S. Lanteri); Roma, S. Pietro FBF (R. Serdoz, E. Bruno); San Felice a Cancello (C. De Matteis, C. Campagnuolo); San Giuseppe Vesuviano (M.A. Ammirati, V.M. Corrado); Arco (M.A. Amado Eleas); Aversa (L. Fattore); Avezzano (C. Ippoliti); Conegliano (G. Turiano); Feltre (C. Piergentili); Gallarate (S.I. Caico); Genova, S. Martino (F. Chiarella); Napoli, S.G. Bosco (P. Capogrosso); Pavia, ICS Maugeri SPA Società Benefit (M. Perotti); Pescia (S. Di Marco); Pozzuoli (G. Sibilio); Sessa Aurunca (L. Di Lorenzo); Taranto (A. Aurelio); Vicenza (A.B. Ramondo); Bari, Policlinico (D. Zanna); Castelfranco Veneto (C. Cernetti); Giugliano in Campania (G. Napolitano); Imola (S. Negroni); Latina (N. Alessandri); Mestre, Dell'Angelo (F. Rigo); Molfetta (F. Giusti); Nuoro (G. Casu); Peschiera Del Garda (A. Vicentini); Policoro (G. Calculli); Pomezia (M.S. Fera); Vittoria (G.V. Lettica); Volterra (G. Vagheggini); Bergamo (A. Pitì); Caserta, Villa del Sole (A. Porfidia); Ciriè (A. Di Leo); Ivrea (A. Ravera); Licata (E. Ciotta); Mirano (S. Saccà); Napoli, AORN Cardarelli, Cardiologia Generale c/Riabilitazione (O. Silvestri); Piombino (S. Isidori); S. Omero (P. Natali); San Bonifacio (M. Anselmi); San Donato Milanese, IRCCS, Cardiologia c/UTIC (L. Testa); Sesto San Giovanni (A. Antonelli); Sondrio (E. Tavasci); Telese (G. Furgi); Teramo (A. Lavorgna); Treviso (N. Gasparetto); Udine (T. Bisceglia).
